# Circulating MiRNA-21-enriched extracellular vesicles promote bone remodeling in traumatic brain injury patients

**DOI:** 10.1038/s12276-023-00956-8

**Published:** 2023-03-03

**Authors:** Ze Lin, Yuan Xiong, Yun Sun, Ruiyin Zeng, Hang Xue, Yiqiang Hu, Lang Chen, Guodong Liu, Adriana C. Panayi, Wu Zhou, Faqi Cao, Fei Gao, Bobin Mi, Guohui Liu

**Affiliations:** 1 Department of Orthopaedics, Union Hospital, Tongji Medical College, Huazhong University of Science and Technology, Wuhan, 430022 P. R. China; 2 Department of Neurosurgery, Union Hospital, Tongji Medical College, Huazhong University of Science and Technology, Wuhan, 430022 P. R. China; 3 Medical Center of Trauma and War Injuries, Daping Hospital, Army Medical University, Chongqing, 400042 P. R. China; 4 Department of Plastic Surgery, Brigham and Women’s Hospital, Harvard Medical School, Boston, MA 02152 USA

**Keywords:** Trauma, Biological therapy

## Abstract

Fracture combined with traumatic brain injury (TBI) is one of the most common and serious types of compound trauma in the clinic and is characterized by dysfunction of cellular communication in injured organs. Our prior studies found that TBI was capable of enhancing fracture healing in a paracrine manner. Exosomes (Exos), as small extracellular vesicles, are important paracrine vehicles for noncell therapy. However, whether circulating Exos derived from TBI patients (TBI-Exos) regulate the prohealing effects of fractures remains unclear. Thus, the present study aimed to explore the biological effects of TBI-Exos on fracture healing and reveal the potential molecular mechanism. TBI-Exos were isolated by ultracentrifugation, and the enriched miR-21-5 p was identified by qRT‒PCR analysis. The beneficial effects of TBI-Exos on osteoblastic differentiation and bone remodeling were determined by a series of in vitro assays. Bioinformatics analyses were conducted to identify the potential downstream mechanisms of the regulatory effect of TBI-Exos on osteoblasts. Furthermore, the role of the potential signaling pathway of TBI-Exos in mediating the osteoblastic activity of osteoblasts was assessed. Subsequently, a murine fracture model was established, and the effect of TBI-Exos on bone modeling was demonstrated in vivo. TBI-Exos can be internalized by osteoblasts, and in vitro, suppression of *SMAD7* promoted osteogenic differentiation, whereas knockdown of miR-21-5 p in TBI-Exos strongly inhibited this bone-beneficial effect. Similarly, our results confirmed that preinjection of TBI-Exos led to enhanced bone formation, whereas knockdown of exosomal miR-21-5 p substantially impaired this bone-beneficial effect in vivo.

## Bone fracture: faster healing after traumatic brain injury

Extracellular vesicles enriched with a particular microRNA released following traumatic brain injury (TBI) help fractures heal faster and could inform treatments for skeletal disorders. Patients with TBI and associated fractures experience shorter fracture recovery times than patients with fractures only, but precisely why is unclear. Ze Lin at Huazhong University of Science and Technology, Wuhan, China, and co-workers examined molecules released during recovery in samples taken from patients with TBI and fractures, those with fractures only, and mouse models with femur fractures. They found that exosomes (extracellular vesicles that transport metabolites including microRNA) released following TBI are enriched with miRNA-21-5 p, which enhances differentiation of bone-forming cells and bone repair. No miRNA-21-5 p enrichment occurred in patients with fractures only. Knocking out exosomal miRNA-21-5 p impaired bone repair in mice, while injecting exosomes from TBI patients boosted bone formation.

## Introduction

Bone fracture is one of the most common injuries encountered in compound trauma and may lead to morbidity and a long rehabilitation period^1^. Fracture combined with traumatic brain injury (TBI) is one of the most common and serious types of compound trauma in the clinic and is characterized by dysfunction of cellular communication in injured organs^2^. Interestingly, patients with fracture and TBI have long been observed to have an obviously shorter healing period than isolated fracture patients. Prior studies have reported a potential relationship between TBI and rapid fracture healing^3–5^. However, the potential mechanisms underlying this interesting phenomenon remain unknown.

Exosomes (Exos), a promising type of bioactive nanomaterial, are capable of exerting their regulatory function through the release of active cargo, including exosomal miRNA, which can bind with target mRNAs to modulate their expression rate and degradation with rare interference by internal and external environmental factors^6^. Currently, the regulatory role of Exos in bone homeostasis has attracted considerable attention^7^. A recent study reported that Exos derived from endothelial cells are capable of reversing osteoporotic activity in vitro and in vivo with highly efficient bone targeting and identified a potential application of Exos as a bone-targeting nanomedicine in reversing bone resorption disorders^8^. Similarly, a prior study indicated that osteoclasts were capable of directly affecting osteoblastic differentiation via delivery of exosomal miR-214-3 p, suggesting that knockdown of miR-214-3 p in osteoclasts may be an effective way to ameliorate bone-impaired skeletal disorders^9^.

MiR-21-5 p is a well-known osteogenic-related miRNA in the regulation of bone remodeling^10^. A recent study reported that exosomal miR-21-5 p derived from growth hormone-secreting pituitary adenoma induced bone formation in vitro and increased trabecular number in vivo^11^. Furthermore, Dai et al. found that miR-21-5 p expression was significantly elevated from the acute to the chronic phase of TBI, and neuronal Exos enriched with miR-21-5 p were able to attenuate trauma-induced nerve injury^12^.

In addition, in this study, we sought to elucidate the beneficial role of TBI in fracture healing with a particular focus on TBI-Exos. We demonstrate that miR-21-5 p is markedly enriched in TBI-Exos, which can be delivered to hMSCs, wherein it binds with *SMAD7* to enhance osteoblastic differentiation and fracture healing.

## Materials and methods

### hMSC isolation, culture, and transfection

First, we collected bone marrow from 10 patients aged 20–30 years with simple fractures without TBI for hMSC extraction. Five milliliters of bone marrow from each patient was extracted under sterile conditions, diluted with an equal volume of PBS, and then centrifuged at 1500 rpm for 10 min at room temperature. Next, the fat layer was discarded, and the diluted marrow solution was slowly added to an equal volume of Ficoll separation solution (1.077 g/mL) and centrifuged at 2500 rpm for 30 min at room temperature. Then, the mononuclear cells were suspended in phosphate buffered saline (PBS) and centrifuged at 1500 rpm for 10 min at room temperature. Subsequently, the cells were suspended in special medium for hMSCs (#HUXMA-9001c, Cyagen, Guangzhou, China) and cultured in an incubator at 37 °C and 5% CO_2_. When the cell growth reached 80%–90% confluence, trypsin-EDTA solution (0.25%:0.02%) was used for cell collection. Small interfering RNAs (siRNAs) were designed and constructed by GenePharma company (Soochow, China). The silencing efficiency of the target gene was verified by qRT‒PCR and western blotting analysis.

### Exo isolation and examination

First, the collected samples were moved to a centrifuge tube and centrifuged for 30 min at 2000 × g and 4 °C. Then, the obtained cell supernatant was moved to a new centrifuge tube and centrifuged for 45 min at 10,000 × g and 4 °C. After the large extracellular vesicles were removed, the supernatant was collected. Next, filtration was performed using a 0.45 μm membrane, and the filtered fluid was collected and centrifuged for 70 min at 100,000 × g at 4 °C. Finally, the supernatant was removed and resuspended in 100 μL of precooled PBS. 10 μL of Exos was used for measuring the particle size by nanoparticle tracking analysis (NTA), 10 μL of Exos was used for exosomal biomarker analysis by Nano-Flow-Cytometry (Nano-FCM), and 20 μL of Exos was used for exosomes shape detection by scanning electron microscopy (SEM). The remaining Exos were stored at −80 °C for subsequent experiments.

### Exos uptake assay

First, the Exo dye PKH26 (Sigma-Aldrich, MO, United States) was used for detection of the purified Exos in hMSCs. Next, 20 mL of PBS was used to wash the Exos twice. The hMSC uptake of the labeled Exos was captured by measuring the immunofluorescence (red indicates Exos, and blue indicates the cell nucleus).

### qRT‒PCR analysis

First, TRIzol (Invitrogen, Shanghai) was used to extract total RNA, and a Verso cDNA Synthesis Kit (Thermo Fisher Scientific, Shanghai) was used to reverse-transcribe the RNA according to the kit instructions. The SeraMir Exosome RNA Purification Kit (System Biosciences, Mountain View, USA) was used to extract miRNAs, and a TaqMan microRNA assay kit (Applied Biosystems, Foster City, USA) was utilized for cDNA synthesis. A StepOne Real-Time PCR system (Life Technologies, Carlsbad, CA, USA) was used for RT-qPCRs, and each sample was added to three multiple wells. The 2^−ΔΔCt^ method was used to calculate the relative mRNA expression. The primers were as follows: miR-21-5 p, forward primer: 5’-GCCCGCTAGCTTATCAGA CTGATG-3’, reverse primer: 5’-GTGCAGGGTCCGAGGT-3’; GAPDH, forward primer: 5’-CCGTTGAATTTGCCGTGA-3’; reverse primer: 5’-TGATGACCCTTTTGGCTCCC-3’.

### Western blotting

First, the total cell protein extraction reagent was added to extract the protein, and the protein concentration of the sample was measured using a BCA protein concentration determination kit. Then, SDS‒PAGE electrophoresis was performed to determine the loading amount according to the sample concentration, ensuring that the total protein loading amount of each sample was 40 μg. The PVDF membrane was activated with methanol for 3 min before use. The membrane transfer time was adjusted according to the molecular weight of the target protein. Next, antibody incubation was carried out, and the transferred membrane was added to the blocking solution at room temperature for sealing for 1 h. Then, the blocking solution was removed, and the primary antibody was added to the solution at 4 °C and incubated overnight with anti-*collagen I* (#ab34710, Abcam, Cambridge, UK, 1/5000), anti-*Runx2* (#ab76956, Abcam, Cambridge, UK, 1/3000), anti-*osteocalcin* (#ab133612, Abcam, Cambridge, UK, 1/5000), anti-*SMAD7* (#ab216428, Abcam, Cambridge, UK, 1/1000), and anti-*β-actin* (#ab8245, Abcam, Cambridge, UK, 1/6000). The diluted primary antibody was recovered, and the membrane was washed three times with TBST for 5 min each time. The diluted secondary antibody was then added, and the membrane was incubated at room temperature for 30 min and washed with TBST on a shaker at room temperature 4 times (5 min each time). Finally, chemiluminescence detection was carried out.

### EdU assay

The hMSC proliferation rate was assessed using the EdU assay kit (RiboBio, Guangzhou, China) as described in a prior study^13^. In brief, hMSCs were seeded into a 24-well plate at a cell density of 2.0 × 10^4^ cells/well and cultured for 24 h before the addition of EdU (50 mM). Then, the Apollo and DNA stains were added to the medium. Finally, EdU assay images were captured using fluorescence microscopy (Carl Zeiss, Jena, Germany).

### ALP and alizarin red staining

An alkaline phosphatase color development kit (#C3206; Beyotime Biotechnology, Shanghai, China) was used to perform ALP staining of hMSCs. BCIP/NBT substrate was used to treat cells for 7 days. Colorimetric changes were analyzed using a charge-coupled microscope, and absorbance was measured at 405 nm. For alizarin red staining, hMSCs were continuously cultured in 24-well plates in osteogenic medium (#MUBMX-03011-440, Cyagen, Guangzhou, China) for 21 days to enhance osteogenesis. Red mineralized nodules were analyzed with a charge-coupled device microscope. Absorbance was measured at 570 nm.

### Luciferase assay

The *SMAD7* 3’-UTR was designed and inserted into the pMIR-report plasmid. For the luciferase reporter assays, hMSCs were cultured and transfected with 0.5 μg firefly luciferase reporter plasmid, 0.5 μg vector, and equal amounts of antagomiR-NC or antagomiR-21-5 p using Lipofectamine 3000 (Invitrogen). hMSCs were analyzed using luciferase assay kits (Promega, Madison, WI, USA).

### Ethics consent

The patient studies were approved by the Committees of Clinical Ethics in the Union Hospital, Tongji Medical College, Huazhong University of Science and Technology (HUST), and informed consent was obtained from all participants (UHCT-IEC-SOP-016-02-01, approval date: 10 Nov 2020). The animal experiments were approved by the Animal Care and Use Committee of Tongji Medical College, HUST.

### Animal administration and histopathological examination

The murine fracture model was constructed as described in a prior study^14^. Briefly, C57BL/6 J male mice were purchased from the Center of Experimental Animal, Tongji Medical College, HUST. Pentobarbital sodium (80 mg/kg body weight, 1%) was used for animal anesthesia. Then, a posterolateral incision was made and blunt separation was performed to expose the femur. Next, a diamond disk was used to make a femoral midsection fracture, and the fracture site was fixed using 0.6 mm diameter intramedullary needles. Local injection of PBS (100 µL) at the fracture site was set as the control group, and mice that received 100 µL of Ctr-Exos (100 µg/mL), TBI-Exos (100 µg/mL), or TBI-Exos+AntagomiR-21-5 p (100 µg/mL) were set as the treatment groups. The animals were grouped according to their needs in each experiment, and each group contained 5 mice. All local injections were performed at Day 0, Day 4, and Day 7 post-injury. On Day 14, half of the mice were sacrificed, and bone samples were collected for histological analysis. Briefly, femurs were fixed with 4% paraformaldehyde overnight and demineralized for 2 weeks. Paraffin-embedded tissue samples (7 µm thick) were prepared and subjected to Alcian Blue/hematoxylin-eosin/orange G (ABHEOG) staining and CD31 and Ki67 staining as previously described^15,16^.

### X-ray images

Small animal X-ray examination was carried out at the Central Laboratory of Union Hospital, Tongji Medical College, HUST. Briefly, animals were anesthetized with 1% pentobarbital sodium (80 mg/kg body weight) and imaged using an in vivo FX PRO imaging system (Bruker, Karlsruhe, Germany) with a one-minute exposure time.

### Microcomputer tomography (Micro-CT) examination

Mice were scanned using the Bruker SkyScan 1176 scanner with the following parameters: 2400 views, five frames/view, 37 kV, and 121 mA. The samples were rotated to 180° with a rotation step of 0.5°, and a 0.5 mm thick Al filter was applied. Image pixel size was set to 9 μm. The threshold level for the evaluation of bone volume was 50–120. Three-dimensional construction was performed using CT-Vox software (2.1 version, Bruker, Karlsruhe, Germany). Bone volume (BV), trabecular volume (TV), BV/TV, and bone mineral density (BMD) were assessed with CTAN software (1.12 version, Bruker, Karlsruhe, Germany).

### Statistical analysis

All data are shown as the means ± SDs. GraphPad Prism 8.0 (GraphPad Software, CA, USA) was used for the statistical analysis. Statistical analysis was performed using Student’s t test (two groups) or ANOVA with Tukey’s post hoc test (over two groups). *P* < 0.05 was the significance threshold.

## Results

### TBI-Exos accelerate fracture healing

**Fig. 1:**
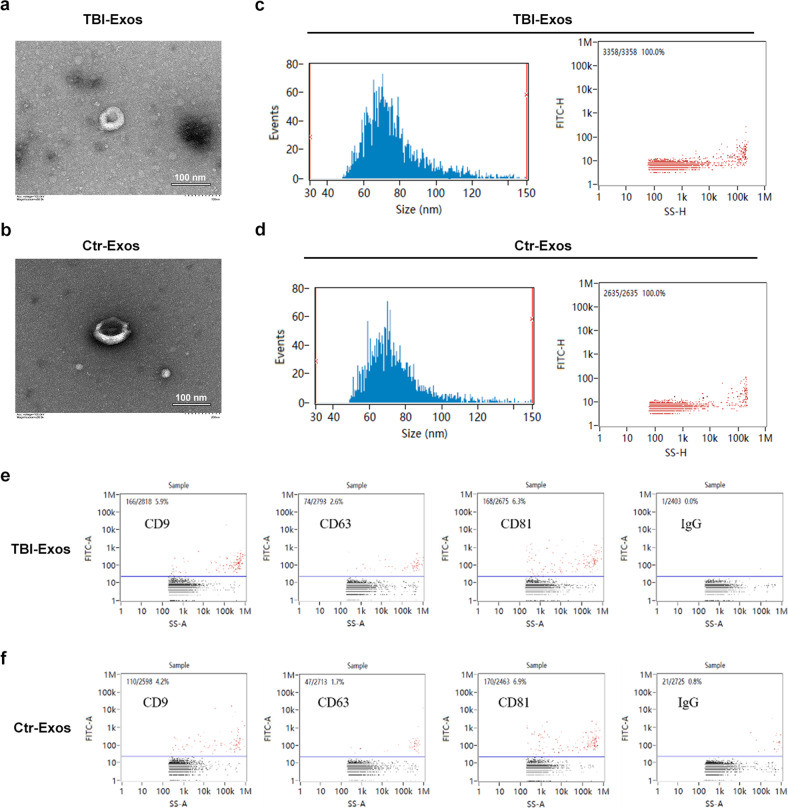
The characteristics of Ctr-Exos and TBI-Exos. **a**, **b** TEM images of Ctr-Exos and TBI-Exos, scale bar: 100 nm. **c**, **d** The size of these nanoparticles was measured by NTA. **e**, **f** The surface markers of Exos, including CD9, CD63, and CD81, were detected using NanoFCM.

**Fig. 2:**
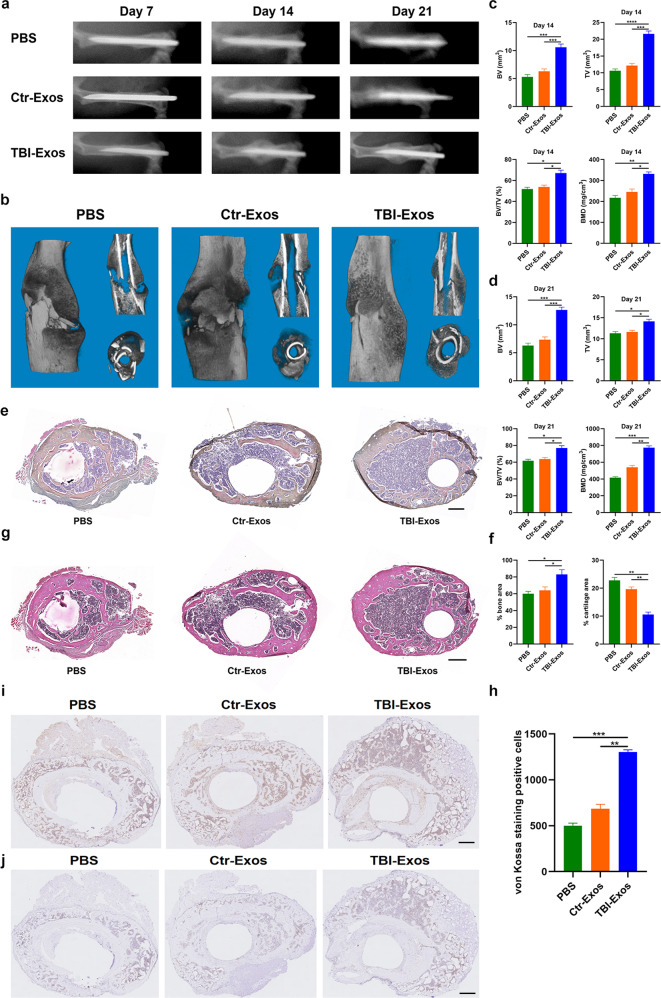
TBI-Exos accelerate fracture healing in vivo. **a**, **b** The healing processes in the mouse model with different treatments were assessed by X-ray **a** and micro-CT analysis **b**. **c**, **d** The CT Analyzer was used to measure the BV, TV, BV/TV and BMD results of mice on Day 14 and Day 21 after fracture. **e**–**h** The ABHEOG and Von Kossa staining results of mice on Day 21 after fracture, scale bar: 200 μm. **i**, **j**, CD31 and Ki67 immunohistochemistry of the callus in different groups, scale bar: 500 μm. Data are the mean ± SD of triplicate experiments. *p < 0.05, **p < 0.01, ***p < 0.001.

First, we assessed whether the isolated vesicles from serum were Exos using TEM, NTA, and NanoFCM analysis. The TEM results showed that the vesicles had cup or sphere shapes, which is consistent with a prior study (Fig. 1a, b)^17^. Furthermore, the NTA results indicated that the diameters of these vesicles ranged from 30 nm to 150 nm, which is consistent with a prior study (Fig. 1c, d)^17^. In addition, the NanoFCM results verified that these vesicles contained exosomal surface markers, including CD9, CD63 and CD81 (Fig. 1e, f). All these results demonstrated that the isolated vesicles were Exos. Next, we examined the therapeutic effect of these two kinds of Exos on murine fracture healing. The mice were randomly divided into three groups according to the different treatments (PBS, Ctr-Exo, and TBI-Exo groups). X-ray and micro-CT examinations were performed to assess the process of murine fracture healing, and the TBI-Exo group showed markedly larger callus formation with a narrower fracture gap than the PBS group and Ctr-Exo group (Fig. 2a, b). The CT analysis results indicated that mice in the TBI-Exo group had a higher BV, TV, BV/TV and BMD than the mice in the PBS group and Ctr-Exo group (Fig. 2c, d). After fixation with 0.5% paraformaldehyde, bone samples were collected for ABHEOG and Von Kossa staining, and the TBI-Exo-treated mice exhibited better bone formation performance and less cartilage formation than those in the PBS group and Ctr-Exo group (Fig. 2e, h). CD31 and Ki67 immunohistochemistry indicated a positive effect on angiogenesis and proliferation of TBI-Exos (Fig. 2i, j, and Supplementary Fig. 1). Moreover, the mechanical properties of the fractured bone were measured by three-point bending, which also indicated that the mice in the TBI-Exo group had better bone formation performance (Supplementary Fig. 2).

### TBI-Exos promote proliferation and osteogenic differentiation in vitro

**Fig. 3:**
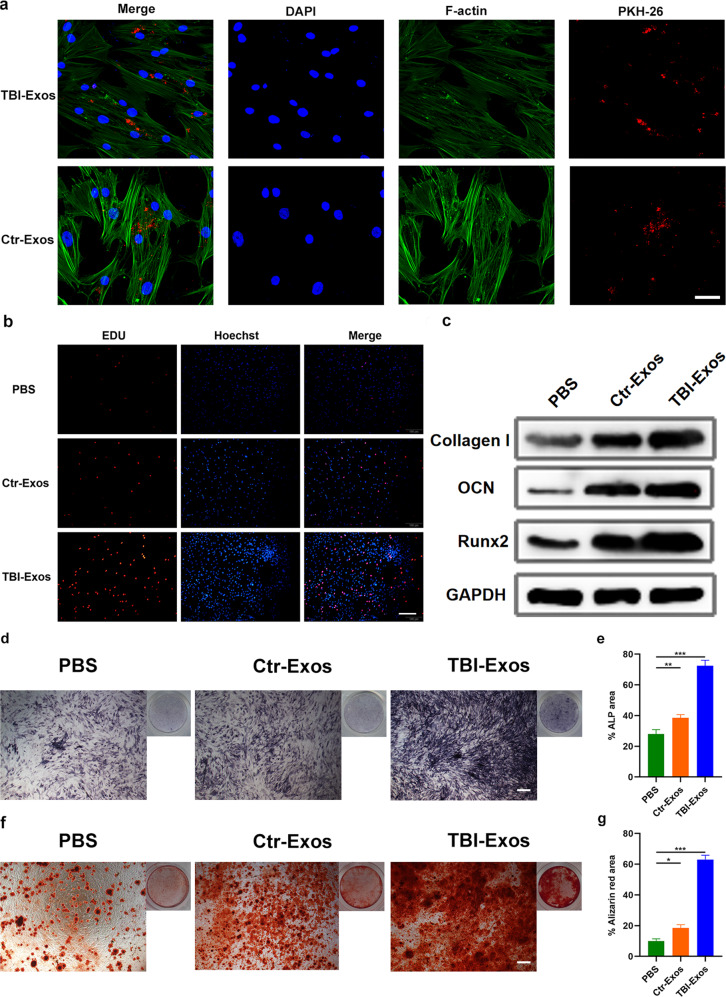
TBI-Exos promote hMSC proliferation and osteogenic differentiation. **a** Uptake assay of Ctr-Exos and TBI-Exos in hMSCs. Scale bar: 50 μm. **b** EdU assay results of hMSCs with different treatments. Scale bar: 50 μm. **c** The levels of osteogenic-related proteins were measured by western blotting. **d**, **e** ALP staining results of hMSCs in the different groups. **f**, **g** Alizarin red staining results of hMSCs in the different groups. Data are the mean ± SD of triplicate experiments. *p < 0.05, **p < 0.01, ***p < 0.001.

Next, we examined whether the two kinds of Exos could be taken up by hMSCs. The Exos were labeled with PKH26 dye (red) and then added to the hMSC medium. The results of this uptake assay suggested that hMSCs were capable of taking up Ctr-Exos and TBI-Exos within 12 h (Fig. 3a). After verifying the uptake ability of hMSCs, we then investigated the effect of these two Exos on the proliferation of hMSCs using an EdU assay. The results showed that TBI-Exos noticeably promoted hMSC proliferation relative to that in the mice in the PBS- and Ctr-Exo-treated groups (Fig. 3b). Furthermore, to test the effect of the two Exos on osteogenic differentiation, we performed western blotting analysis to assess osteogenic-related proteins, including *Collagen I, Runx 2* and *OCN*, among the different groups. The results indicated a high level of these proteins in the TBI-Exo-treated group relative to the other groups (Fig. 3c). Moreover, ALP staining and alizarin red staining were performed to determine the level of extracellular mineral deposition, and the results suggested that TBI-Exos were able to induce enhanced mineral deposition compared with the other treatments (Fig. 3d–g).

### Exosomal miR-21-5p is important for the effect of TBI on fracture healing

**Fig. 4:**
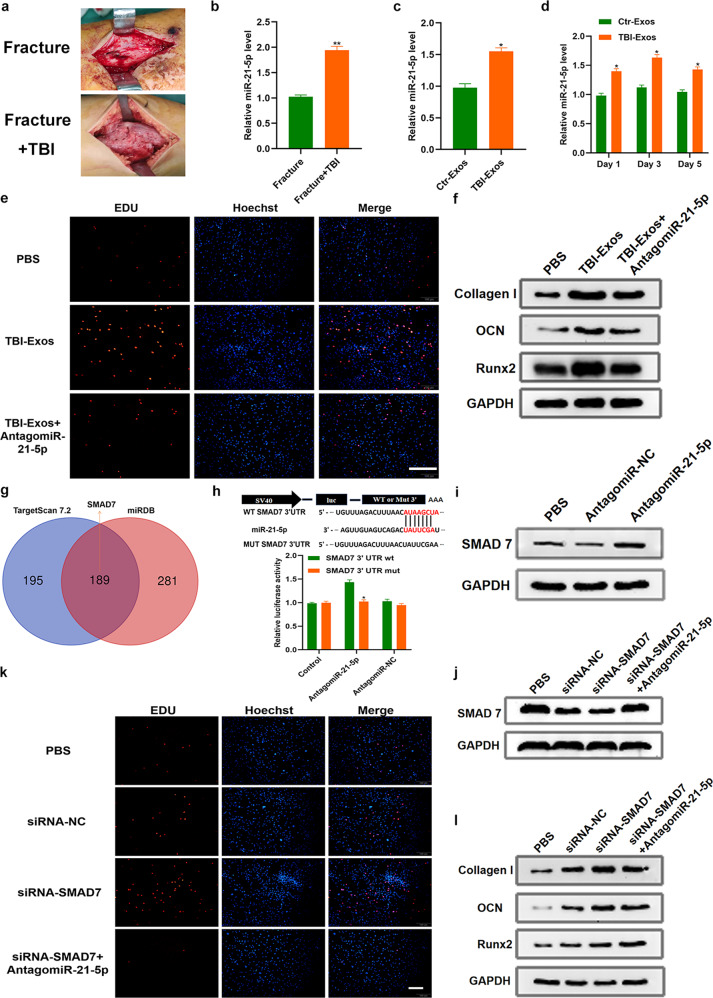
The underlying mechanism of TBI in the regulation of fracture healing. **a** The calluses at the fracture site were more visible in the fracture+TBI group than in the fracture group. **b**, **c** The relative miR-21-5 p level was measured in both serum and Exos using qRT‒PCR analysis. **d** The miR-21-5 p expression of hMSCs in different groups was measured by qRT‒PCR analysis. **e** An EdU assay was performed to assess cell proliferation in the different groups. **f** Western blotting was used to detect osteogenic-related proteins in the different groups. **g** The potential targets of miR-21-5 p were identified using online prediction tools. **h** A luciferase reporter assay was performed to test the interaction of miR-21-5 p and *SMAD7*. **i**, **j** *SMAD7* levels in the different groups were measured by western blotting analysis. **k** The EdU assay was used to evaluate hMSC proliferation. **l** Western blotting was used to measure the expression of osteogenic-related proteins. Data are the mean ± SD of triplicate experiments. *p < 0.05, **p < 0.01, ***p < 0.001.

In the clinic, we found superior healing performance in fracture patients with TBI relative to isolated fracture patients (Fig. 4a). Exosomal miRNA is an essential element of the Exos’ cargo, which has been previously demonstrated to regulate biological processes. Notably, miR-21-5 p is one of the key osteogenic-related miRNAs and has been reported to be enriched in TBI-Exos^18,19^. Thus, we assessed the miR-21-5 p level in both patient serum and Exos. The results showed that miR-21-5 p in both serum and Exos was significantly elevated after TBI (Fig. 4b, c). Furthermore, hMSCs were treated with 50 μg/mL Ctr-Exos or 50 μg/mL TBI-Exos for 1 day, 3 days and 5 days. The qRT‒PCR results suggested that the miR-21-5 p level was significantly higher in the TBI-Exo-treated cells than in the Ctr-Exo-treated cells at the three timepoints (Fig. 4d). Next, to assess the effect of miR-21-5 p on osteoblast proliferation and differentiation, we transfected hMSCs with antagomiR-NC or antagomiR-21-5 p. The qRT‒PCR results suggested efficient suppression of miR-21-5 p expression by antagomiR-21-5 p (Supplementary Fig. 3). Next, we investigated the effect of the different treatments on the proliferation of hMSCs using the EdU assay. The results showed that TBI-Exos noticeably promoted hMSC proliferation, whereas miR-21-5 p inhibition markedly impaired this pro-proliferative effect of TBI-Exos (Fig. 4e). Similarly, the western blotting results indicated that the addition of antagomiR-21-5 p significantly decreased the TBI-Exo-elevated level of osteogenic-related proteins (Fig. 4f). Furthermore, we investigated the potential downstream mechanism of miR-21-5 p. As shown in Fig. 4g, the potential targets of miR-21-5 p were identified using online prediction tools, including TargetScan (version: 7.2, http://www.targetscan.org/vert_72/) and miRDB (http://mirdb.org/), and *SMAD7* was identified as a predicted target gene. Moreover, to test the interaction of miR-21-5 p and *SMAD7*, we performed a luciferase reporter assay, and the results suggested that miR-21-5 p was capable of specifically binding to the region of *SMAD7* mRNA (Fig. 4h). Subsequently, western blotting indicated that miR-21-5 p inhibition obviously led to an elevation in *SMAD7* levels (Fig. 4i). To determine the role of *SMAD7* in the regulation of hMSC proliferation and differentiation, we used SMAD7 siRNA. The qRT‒PCR results verified the high efficiency of SMAD7 siRNA in suppressing *SMAD7* expression, and the addition of antagomiR-21-5 p partially reversed the reduction in *SMAD7* (Fig. 4j). In addition, we assessed the effect of *SMAD7* on the proliferation of hMSCs using an EdU assay, which showed that siRNA *SMAD7* could noticeably promote hMSC proliferation, whereas miR-21-5 p inhibition could partially impair this pro-proliferative effect of siRNA *SMAD7* (Fig. 4k). Similarly, the western blotting results indicated that the addition of antagomiR-21-5 p could significantly decrease the siRNA *SMAD7*-elevated level of osteogenic-related proteins (Fig. 4l).

### Exosomal miR-21-5p accelerates fracture healing in vivo

**Fig. 5:**
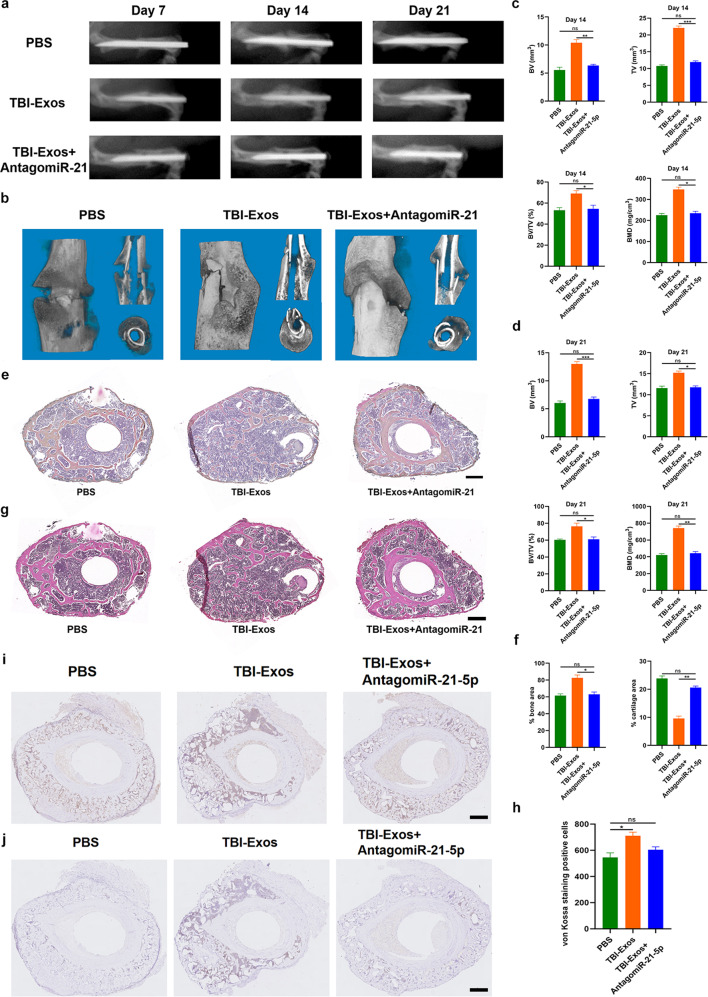
Exosomal miR-21-5 p promotes fracture healing in vivo. **a**, **b** The healing processes in the mouse model with different treatments were assessed by X-ray (**a**) and micro-CT analysis (**b**). **c**, **d** The CT Analyzer was used to measure the BV, TV, BV/TV and BMD results of mice on Day 14 and Day 21 after fracture. **e**, **h** The ABHEOG and Von Kossa staining results of mice on Day 21 after fracture, scale bar: 200 μm. **i**, **j** CD31 and Ki67 immunohistochemistry of the callus in different groups, scale bar: 500 μm. Data are the mean ± SD of triplicate experiments. *p < 0.05, **p < 0.01, ***p < 0.001.

Finally, we assessed the therapeutic effect of exosomal miR-21-5 p on murine fracture healing. The mice were randomly divided into three groups according to the different treatments (PBS, TBI-Exo, and TBI-Exo+antagomiR-21-5 p groups). X-ray and micro-CT examinations were performed to assess the process of murine fracture healing, and the TBI-Exo group showed markedly larger callus formation with a narrower fracture gap than the PBS group, whereas after the addition of antagomiR-21-5 p, the pro-healing effect of TBI-Exos was partially reversed (Fig. 5a, b). The CT analysis results indicated that the mice in the TBI-Exo group had higher BV, TV, BV/TV and BMD than the mice in the PBS group, and the addition of antagomiR-21-5 p partially reversed the effect of TBI-Exos (Fig. 5c, d). After fixation with 0.5% paraformaldehyde, bone samples were collected for ABHEOG and Von Kossa staining. Similarly, the TBI-Exo-treated mice exhibited better bone formation performance and less cartilage formation than the PBS-treated mice, and the addition of antagomiR-21-5 p partially impaired the prohealing effect of TBI-Exos (Fig. 5e–h). Moreover, CD31 and Ki67 immunohistochemistry indicated a positive effect on angiogenesis and proliferation in the TBI-Exo-treated group, and antagomiR-21-5 p could partially reverse this effect (Fig. 5i, j and Supplementary Fig. 4).

## Discussion

Elucidation of the interaction between TBI and fracture, which may provide novel insights for accelerated fracture-healing strategies, is needed. Prior studies have extensively investigated the role of bioactive regulators in bone metabolism, with few studies identifying the relationship between TBI-related exosomal miRNAs and fracture healing^20–22^. In this study, we found that miR-21-5 p is markedly enriched in TBI-Exos compared with Ctr-Exos, and exosomal miR-21-5 p was further demonstrated to accelerate bone remodeling in vitro and in vivo. Furthermore, the suppression of *SMAD7* signaling was assumed to be the underlying mechanism of this prohealing effect. Our findings revealed that the use of nanomaterials combined with miR-21-5 p-mimics may act as a feasible strategy to enhance fracture healing.

Exos are endogenous nanosized vesicles secreted by a wide array of cells into the extracellular microenvironment^23^. Exos represent an important mode of intercellular communication and serve as an ideal carrier for the delivery of active proteins, lipids, and RNA^24,25^. Currently, due to their robust regulation of cellular communication in prokaryotes and eukaryotes, Exos have attracted increasing research interest^26^. Exosomal miRNA is one of the most important regulators in Exos, and many exosomal miRNAs have previously been reported to modulate bone remodeling^27,28^. In our prior study, we demonstrated that exosomal miR-5106 derived from M2 subtype macrophages induces mesenchymal stem cells toward osteoblastic differentiation and promotes fracture healing^29^. Thus, in the present study, we investigated the exosomal miRNA derived from TBI-Exos and sought to elucidate the mechanism underlying the prohealing effect of TBI on fracture healing. Interestingly, our results indicated that TBI-Exos promote osteogenic differentiation of fracture sites and stimulate local angiogenesis. Prior studies have demonstrated that alteration of the microenvironment induced by the enhanced function of bone mesenchymal stem cells could stimulate angiogenesis through intercellular communication^30,31^. We assume that two issues may contribute to the enhanced angiogenesis in this study. On the one hand, TBI-Exos may also be taken up by endothelial cells, and the function of endothelial cells could be enhanced by the release of active cargos from TBI-Exos, thereby promoting angiogenesis in the fracture sites. On the other hand, TBI-Exos directly mediated the cellular functions of hMSCs and angiogenic endothelial cells and regulated communication between them by promoting paracrine effects via exosomal interactions in vitro. In our next study, we will further investigate the relationship between TBI-Exos and the function of vascular endothelial cells and verify the effect of TBI-Exos on vascular endothelial cell tube-formation function and angiogenesis at fracture sites.

Thus, in this study, we examined whether the miR-21-5 p level was elevated in fracture patients with TBI. We found that miR-21-5 p is markedly enriched in serum and Exos from patients with fracture combined with TBI relative to isolated fracture patients. Similarly, elevated miR-21-5 p expression was also found in hMSCs treated with TBI-Exos. We then examined the effect of exosomal miR-21-5 p on fracture healing in a murine fracture model. The in vivo results demonstrated a noticeable promotion of TBI-Exos on mouse femoral fracture healing, and this effect was partially reversed by miR-21-5 p inhibition. Hence, we concluded that exosomal miR-21-5 p is the main underlying mechanism of TBI’s effect on fracture healing.

Osteoblastic differentiation is essential for bone formation as an important step of fracture healing^32^. Impaired osteoblastic differentiation is assumed to be a crucial issue for disrupted bone remodeling^33^. We identified *SMAD7* as a potential downstream target of miR-21-5 p. Prior studies have documented that *SMAD7* serves as an inhibitory signal for the TGF-β/BMP pathway and suppresses bone formation in a negative feedback manner^34^. In this study, we found a distinct role of *SMAD7* in osteoblastic differentiation. When SMAD7 is downregulated, it is no longer capable of restraining the osteoblastic differentiation process, thus resulting in an improved performance of bone remodeling. Furthermore, when miR-21-5 p inhibition treatment was added, the prodifferentiation effect of SMAD7 siRNA was partially reversed. All these results suggested the miR-21-5 p/*SMAD7* axis in the regulation of osteoblastic differentiation.

Our findings have potential application value for the treatment of diseases related to bone formation disorders. The TBI-Exo-mediated delivery of miR-21-5 p with promoting effects on bone mesenchymal stem cells is probably the main contributor to the induction of osteogenic differentiation. Considering the systemic dysfunction of bone remodeling in patients with bone formation disorders, therapeutic interventions targeting TBI-Exos and miR-21-5 p would probably improve bone regeneration in these patients. In this study, we particularly focused on exosomal miR-21-5 p delivery from TBI-Exos to hMSCs. However, Exos deliver other active factors than miRNAs, potentially regulating the target cells. In addition, other regulatory issues, such as angiogenesis and inflammation, may affect bone remodeling and fracture healing. It is necessary to further elucidate the definite factor of TBI-Exos in mediating fracture healing and uncover the changes in the microenvironment of the fracture site and their roles in fracture healing.

## Supplementary information

Supplementary information

## Data Availability

All data from this study are available within the paper.
