# Supplementary material for: Circulating MiRNA-21-enriched extracellular vesicles promote bone remodeling in traumatic brain injury patients
Source: Exp Mol Med. 2023 Mar 3;55(3):587–96. doi: 10.1038/s12276-023-00956-8 (PMC10073188; doi:10.1038/s12276-023-00956-8)
Supplement: Supplementary file 1 — Supplementary information [file 12276_2023_956_MOESM1_ESM.pdf]

Supplementary information

Circulating miRNA-21-enriched Extracellular Vesicles Promote Bone Remodeling in Traumatic Brain Injury Patients

Ze Lin[1#], Yuan Xiong[1#], Ruiyin Zeng[1#], Hang Xue[1], Yiqiang Hu[1], Lang Chen[1], Guodong Liu[2], Adriana C. Panayi[3], Wu Zhou[1], Yun Sun[4], Faqi Cao[1], Fei Gao[1*], Bobin Mi[1*], Guohui Liu[1*]

[1]Department of Orthopaedics, Union Hospital, Tongji Medical College, Huazhong University of Science and Technology, Wuhan 430022, P. R. China

[2]Medical Center of Trauma and War Injuries, Daping Hospital, Army Medical University, Chongqing 400042, P. R. China

[3]Department of Plastic Surgery, Brigham and Women's Hospital, Harvard Medical School, Boston, MA 02152, USA

[4]Department of Neurosurgery, Union Hospital, Tongji Medical College, Huazhong University of Science and Technology, Wuhan 430022, P. R. China

Correspondence to: mibobin@hust.edu.cn (Bobin Mi), liuguohui@hust.edu.cn (Guohui Liu)

Ze Lin, Yuan Xiong and Ruiyin Zeng contributed equally to this work.

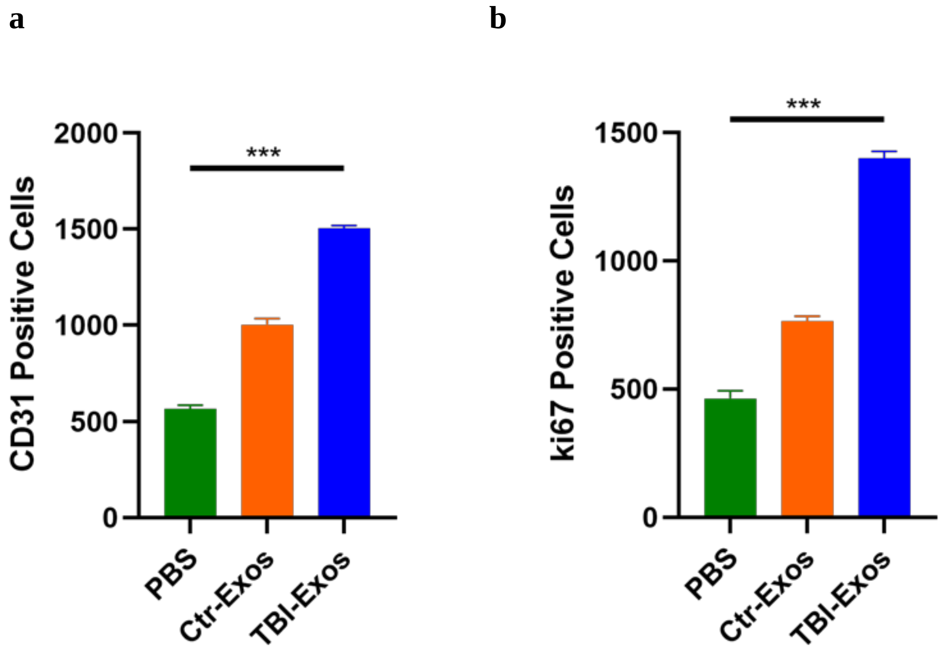

Supplementary Fig. 1. The statistical results of CD31 (a) and Ki67 (b) analysis in the different groups.

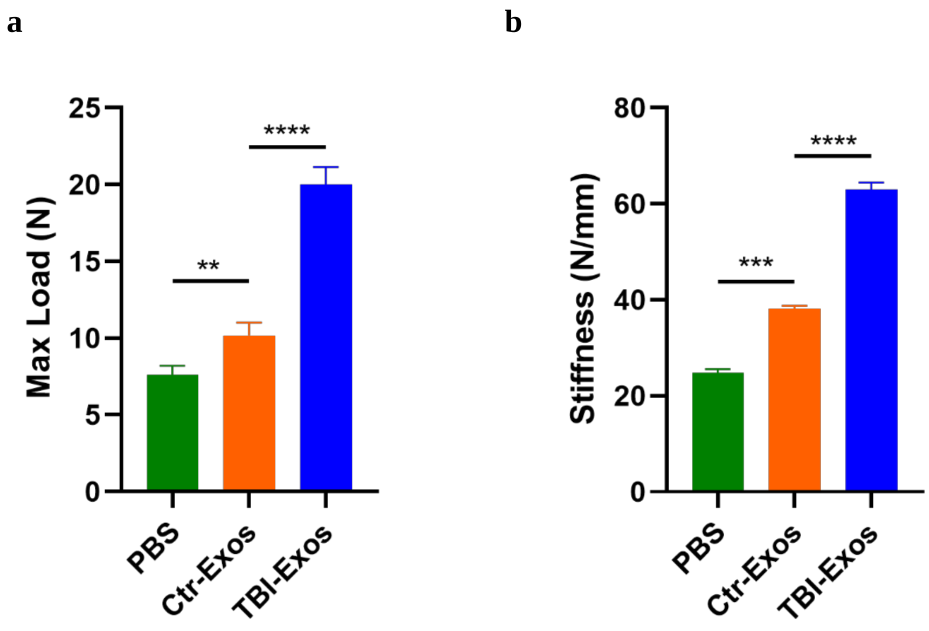

Supplementary Fig. 2. The physical strength of fracture bone, measured by three-point bending. (a) Maximum load to failure, (b) Stiffness.

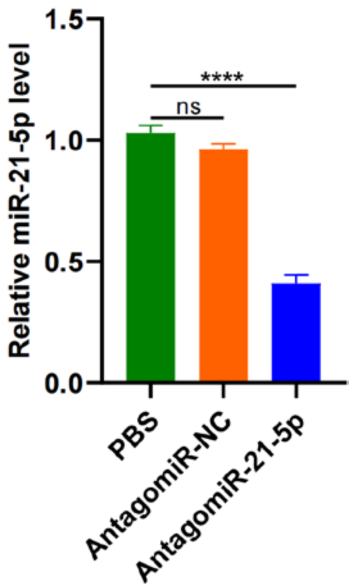

Supplementary Fig. 3. Relative miR-21-5p level of hBMSCs in the different groups was measured by qRT-PCR analysis.

a **b**

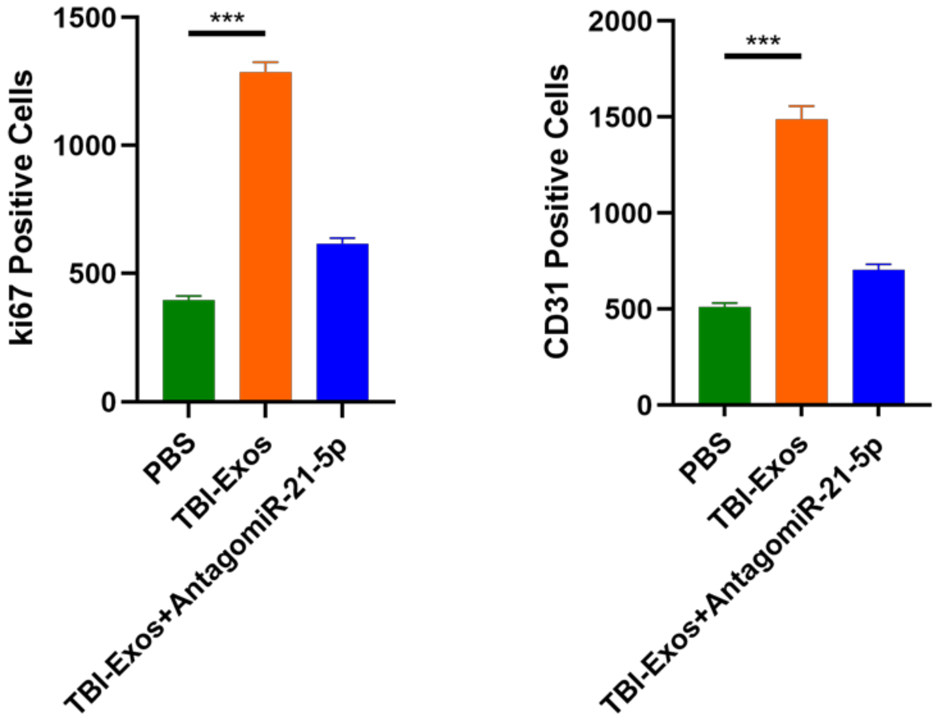

Supplementary Fig. 4. The statistical results of CD31 (a) and Ki67 (b) analysis in the different groups.
